# Age-related immune response disparities between adults and children with severe COVID-19: a case–control study in China

**DOI:** 10.3389/fmicb.2025.1525051

**Published:** 2025-02-04

**Authors:** Hongliang Chen, Yuan Li, Liping Yuan, Fen Liu, Qian Sun, Qingkai Luo, Yefei Lei, Yinglan Hou, Jiayan Li, Liang Cai, Shixing Tang

**Affiliations:** ^1^Department of Epidemiology, School of Public Health, Southern Medical University, Guangzhou, China; ^2^Department of Clinical Microbiology Laboratory, The First People's Hospital of Chenzhou, Chenzhou, China; ^3^Wenzhou Center for Disease Control and Prevention, Wenzhou, Zhejiang, China; ^4^Hunan Provincial Center for Disease Control and Prevention, Hunan, China

**Keywords:** COVID-19, cytokine, cytokine storm, inflammatory response, case–control study

## Abstract

**Background:**

Elucidation of immune response differences is critical for uncovering underlying mechanisms and developing potential intervention measures among adults and children with COVID-19.

**Methods:**

In this retrospective study, we analyzed serum biochemical markers and cytokine profiles among adults and children with COVID-19 in the First People’s Hospital of Chenzhou in Hunan, China from 1 December 2022 to 13 February 2023. A case–control study was conducted using propensity score matching (PSM) to mitigate possible confounding factors.

**Results:**

The significant differences observed included lymphocyte exhaustion, an increased neutrophil-to-lymphocyte (NEU/LYM) ratio, high levels of C-reactive protein (CRP), and a cytokine storm, characterized by high levels of Th1 proinflammatory cytokines, including interleukin 1β (IL-1β), IL-6, IL-8, interferon type I (IFN-*γ*), and tumor necrosis factor (TNF-*α*) in the lung among severe adult COVID-19 patients. Additionally, systemic immune responses were observed in children with COVID-19.

**Conclusion:**

Significant differences in immune responses between adults and children with COVID-19 highlight the different mechanisms and potential intervention measures of COVID-19.

## 1 Introduction

The emergence of severe acute respiratory syndrome coronavirus 2 (SARS-CoV-2) in late 2019 and the pandemic of coronavirus disease 2019 (COVID-19) highlighted the importance of global health preparedness and response system (Cucinotta and Vanelli, 2020) and the vulnerabilities of the existing systems particularly in low- and middle-income regions (Rosenthal and Waitzberg, 2023). The spectrum of COVID-19 clinical presentations is broad, ranging from asymptomatic infection, mild/moderate disease to severe cases (Contini et al., 2023). Severe COVID-19 patients may progress to acute respiratory distress syndrome (ARDS), and, in some cases, fatal outcomes, especially in elderly people and those with compromised immune systems (Chen et al., 2021; Hellman et al., 2024).

In general, SARS-CoV-2 infection is associated with abnormal immune responses (Mathew et al., 2020), including reduction of lymphocytes and increase of neutrophils in peripheral blood in adult COVID-19 patients (Jia et al., 2024) as well as heightened levels of proinflammatory cytokines with an initial decrease of interferon type I (IFN-I) (Ramasamy and Subbian, 2021). The overactive inflammation and inadequate early IFN response to SARS-CoV-2 infection can facilitate the prognosis of COVID-19 (Mehta et al., 2020), and cause serious complications (Lucas et al., 2020). However, different from adult COVID-19 patients, children usually rapidly activate innate immunity upon viral invasion and typically have milder symptoms (Dong et al., 2020), less reduction of peripheral blood lymphocytes (Ma et al., 2021), and weak cytokine storms (Yin et al., 2024). In addition, children often produce a focused antibody response primarily against the spike protein of SARS-CoV-2, with minimal IgG antibodies against viral nucleocapsid protein (Weisberg et al., 2021).

Previous studies have explored immune response differences between adults and children in various respiratory infections (Garofalo et al., 2005), such as infections with influenza viruses and respiratory syncytial virus (RSV). Children with RSV infection often demonstrate more robust IFN-I responses compared to adults, which contributes to distinct disease outcomes (Hijano et al., 2019). Similarly, adults with influenza virus infections usually display more pronounced inflammatory cytokine activity (Bahadoran et al., 2016). These findings highlight the age-related immune response variations in viral infections including SARS-CoV-2 infection in COVID-19 patients. Understanding the age-related immune disparities is critical for identifying tailored interventions and therapeutic approaches.

In December 2022, China adjusted its stringent control strategies, leading to outbreaks of COVID-19 in China (Lu et al., 2023), which provided us with the materials to investigate age-related immune response disparities between adult and pediatric severe COVID-19 patients in real-world settings. We collected bronchoalveolar lavage fluid (BALF) samples and blood in both the progressive and recovery stages of COVID-19. We analyzed cytokine profiles among adults and children with COVID-19 by using a case–control study design. Significant differences were identified in immune responses between adults and children with COVID-19, indicating the different mechanisms and potential intervention measures of COVID-19.

## 2 Materials and methods

### 2.1 Study participants

**Figure 1:**
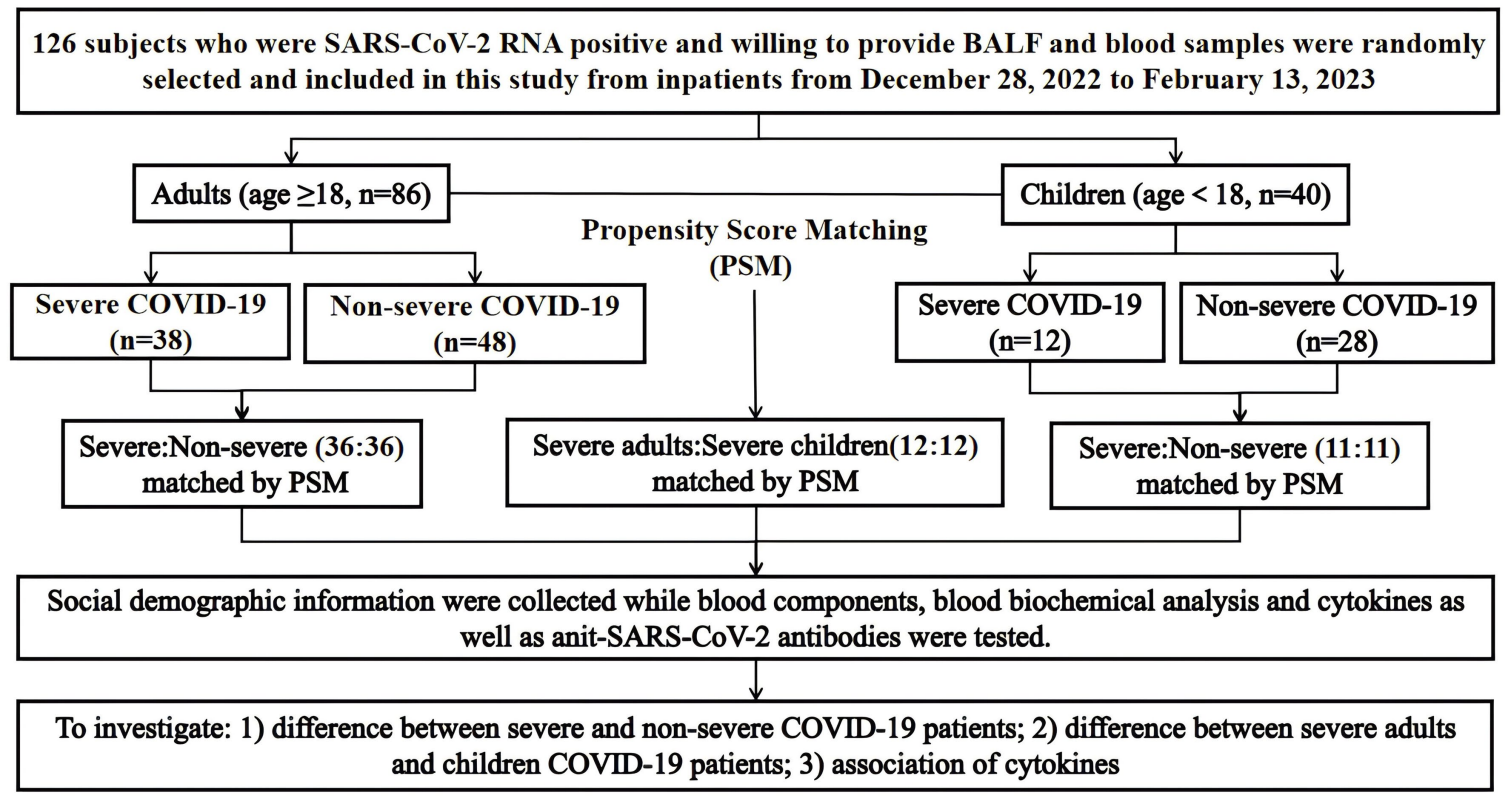
Study design and its flow diagram.

A total of 126 COVID-19 patients were enrolled in the study from the inpatients at the First People’s Hospital of Chenzhou in Hunan Province, China, from 28 December 2022 to 13 February 2023. Among them, 50 severe cases and 76 non-severe cases were diagnosed according to the guidelines for diagnosing and treating SARA-CoV-2 infection (version 10) issued by the National Health Commission of China (National Health Commission of the People’s Republic of China, 2023). Specifically, adults with COVID-19 were defined as non-severe if they met one of the following criteria: (1) absence of COVID-19 symptoms, such as shortness of breath, fever, cough, sore throat, fatigue, headache, muscle pain, nausea, vomiting, diarrhea, taste and smell loss; (2) presence of COVID-19-related symptoms without dyspnea; (3) respiratory rate (RR) < 30 breaths/min and oxygen saturation > 93% in the resting state; and (4) characteristic imaging manifestations of COVID-19 pneumonia. The severe adult COVID-19 patients are those with (1) shortness of breath, RR ≥ 30 breaths/min; (2) oxygen saturation ≤ 93% in the resting state; (3) arterial partial pressure of oxygen (PaO_2_)/inspired oxygen concentration (FiO_2_) ≤ 300 mmHg; (4) pulmonary infiltration >50%; and (5) presence of respiratory failure, septic shock, or multiple organ dysfunction. The criteria for diagnosis of non-severe children COVID-19 patients are (1) absence of COVID-19 symptoms, such as shortness of breath, fever, cough, sore throat, fatigue, headache, muscle pain, nausea, vomiting, diarrhea, taste, and smell loss; (2) presence of COVID-19-related symptoms without dyspnea; (3) persistent high fever for more than 3 days, cough or shortness of breath, but with the normal respiratory rate (RR), that is, RR < 60 breaths/min for children less than 2 months old, or RR < 50 breaths/min in the children of 2–12 months old, or RR < 40 breaths/min in those of 1–5 years old, or RR < 30 breaths/min for more than 5-year-old children, oxygen saturation > 93% in the resting state; and (4) characteristic imaging manifestations of COVID-19 pneumonia. The criteria for severe children COVID-19 patients included (1) high fever or persistent high fever for more than 3 days; (2) shortness of breath, that is, RR ≥ 60 breaths/min for less than 2 months old children, or RR ≥ 50 breaths/min for children of 2–12 months old, or RR ≥ 40 breaths/min for those of 1–5 years old, or RR ≥ 30 breaths/min for more than 5-year-old children except for cases with fever or crying; and (3) oxygen saturation ≤ 93% in the resting state. In addition, all the participants were hospitalized patients, and SARA-CoV-2 positive by quantitative reverse transcription-polymerase chain reaction (qRT-PCR) on throat swabs. We collected social demographic information and medical data including clinical symptoms and signs, clinical laboratory results, such as blood tests, liver and kidney function analysis, and myocardial biomarkers from the inpatient medical record system upon admission. All the patients included in the study were evaluated for co-morbidities, including major chronic diseases, but not other pathogenic infections. However, none of the patients had life-threatening infections that were not related to COVID-19. Informed consent was obtained from the participants, and the ethics committee of Chenzhou First People’s Hospital approved the study (project number: cz2023015). The study design and the details of participant inclusion and exclusion are depicted in Figure 1.

### 2.2 Clinical samples

Both BALF and serum samples that were used to analyze cytokine profiles were collected from patients in progressive stages of COVID-19 on the same day after admission. For each patient, approximately 1.5 mL of BALF samples were collected using fiber optic bronchoscopy. The lung segment was irrigated with saline solution at least 3 times, followed by negative pressure aspiration. At least 30% of the infused fluid was retrieved. The BALF samples were centrifuged, and the supernatant was stored at −80°C. In addition, venous blood was collected from patients in progressive and recovery stages of COVID-19, and the serum samples were stored at −80°C for analysis.

### 2.3 Cytokine detection

Cytokines including IFN-*α*, IFN-*β*, IL-1β, IL-2, IL-4, IL-5, IL-6, IL-8, IL-10, IL-12, IL-17, and TNF-α were detected by using multiplexed microsphere flow immunofluorescence luminescence method and a 12-item cytokine detection kit from Qingdao Realskil Biotechnology Co., Ltd. (Qingdao, China). The normal reference range is as follows: IL-1β ≤ 12.4 pg./mL, IL-2 ≤ 7.5 pg./mL, IL-4 ≤ 8.56 pg./mL, IL-5 ≤ 3.1 pg./mL, IL-6 ≤ 5.4 pg./mL, IL-8 ≤ 20.6 pg./mL, IL-10 ≤ 12.9 pg./mL, IL-12 ≤ 3.4 pg./mL, IL-17 ≤ 21.4 pg./mL, TNF-*α* ≤ 16.5 pg./mL, IFN-γ ≤ 23.1 pg./mL, and IFN-α ≤ 8.5 pg./mL (Liu et al., 2021).

### 2.4 Statistical analysis

Statistical analysis was conducted using R Software (Developed by the R Development Core Team, available under the GNU General Public License). GraphPad Prism 8 Software (Developed by GraphPad Software, Inc.). was used for data visualization. Demographic characteristics were presented for categorical variables whereas continuous data were described using average ± standard variation (SD) or median and interquartile range (IQR). The normal distribution for continuous variables was assessed using the Kolmogorov–Smirnov and Shapiro–Wilk tests. A chi-square test was performed to determine differences in the proportions of categorical variables. Propensity score matching (PSM) was conducted to reduce potential confounding effects. MATCHIT Package in R (Created and maintained by Kosuke Imai and collaborators, available on CRAN). was used to match severe and non-severe COVID-19 cases at a 1:1 ratio using the greedy nearest neighbor method. The overall quality of the matched samples was evaluated by comparing standardized mean differences (SMDs) for the covariates. In contrast, SMDs <0.1 indicate no significant difference in the demographic characteristics (such as age and sex) between the two groups (Lee and Little, 2017). Non-parametric Mann–Whitney U test or *t*-test was used to determine significant differences in blood biochemistry and inflammatory biomarkers between severe and non-severe patients (Mann and Whitney, 1947; Student, 1908). A *p*-value <0.05 was considered statistically significant.

## 3 Results

### 3.1 Demographic and clinical features of participants

The study recruited 126 COVID-19 patients including 86 adults and 40 children. There were 38 severe adult patients and 48 non-severe adult patients as well as 12 severe pediatric patients and 28 non-severe pediatric patients. The significant differences were a higher proportion of chronic diseases, mechanical ventilation, antiviral treatment, and mortality in adults than in children (Supplementary Table S1). To minimize the potential confounding factors, 36 severe adult patients and 36 non-severe adult patients, as well as 11 severe pediatric patients and 11 non-severe pediatric patients, were selected via PSM matching by age, sex, and chronic disease. For the matched adult severe and non-severe COVID-19 patients, the only differences were the proportion of antiviral treatment (41.7% vs. 5.6%) and mortality (25.0% vs. 5.6%). In contrast, no significant difference was found between matched severe and non-severe patients of children (Table 1). In addition, significant differences in the proportion of chronic diseases, mechanical ventilation, antiviral treatment, mortality, and vaccination with the COVID-19 vaccine were also observed between the matched adults and children with severe COVID-19 (Table 1).

**Table 1:** Comparison of baseline information for the enrolled COVID-19 patients through propensity score matching.

Characteristics	Adults with COVID-19^a^		*p-*value^d^	Children with COVID-19^b^		*p-*value^d^	Severe COVID-19 patients^c^		*P-*value^d^
	Severe (*n* = 36)	Non-severe (*n* = 36)		Severe (*n* = 11)	Non-severe (*n* = 11)		Adults (*n* = 12)	Children (*n* = 12)	
Age (years, median, IQR)	59.00(55.00,69.00)	64.00(54.00,71.00)	0.888	3.00(1.00,6.00)	4.00(1.00,7.00)	0.594	57.50 (50.50, 72.25)	4 0.00(0.56, 6.00)	**<0.001**
Sex (*n*)			0.796			1.000			1.000
Male	26(72.2)	25(69.4)		6(54.5)	6(54.5)		7(58.3)	7(58.3)	
Female	10(27.8)	11(30.6)		5(45.5)	5(45.5)		5(41.7)	5(41.7)	
Chronic diseases (*n*, %)			0.796			1.000			**0.001**
Diabetes mellitus	11(30.6)	4(11.1)		0(0)	0(0)		4(33.3)	0(0)	
Hypertension	14(38.9)	8(22.2)		0(0)	0(0)		6(50.0)	0(0)	
Cerebral or cardiac vascular disease	10(27.8)	7(19.4)		0(0)	0(0)		2(16.7)	0(0)	
Malignant tumor	2(5.6)	10(27.8)		0(0)	0(0)		0(0)	0(0)	
COPD	0(0)	2(5.6)		0(0)	0(0)		0(0)	0(0)	
Antiviral treatment (*n*, %)	15(41.7)	2(5.6)	**<0.001**	0(0)	0(0)	1.000	4(33.3)	0(0)	**0.028**
Mechanical ventilation (*n*, %)	5(13.9)	2(5.6)	0.233	0(0)	0(0)	1.000	2(16.7)	0(0)	0.140
Length of hospitalization (days, median, IQR)	12.00(8.00,19.00)	9.50(6.00,17.00)	0.700	7.50(5.75,10.25)	6.00(5.00,6.00)	0.056	15.00(11.25,30.25)	7.00(5.00,10.00)	**<0.001**
Vaccination (*n*, %)			0.752			0.865			**0.000**
Unvaccinated	11(37.9)	7(30.4)		6(60.0)	7(63.6)		0(0)	6(60.0)	
Full vaccination	2(6.9)	1(4.3)		4(40.0)	4(36.4)		0(0)	4(40.0)	
Booster vaccination	16(55.2)	15(65.2)		0(0)	0(0)		10(100)	0(0)	
Outcome (*n*, %)			**0.005**			1.000			1.000
Recovery	27(75.0)	34(94.4)		11(100)	11(100)		12(100)	12(100)	
Death	9(25.0)	2(5.6)		0(0)	0(0)		0(0)	0(0)	

a The adults were matched by age, sex and chronic diseases through propensity score matching with a matching tolerance of 0.3.

b The children were matched by age and sex through propensity score matching with a matching tolerance of 0.5.

c All the subjects were matched by sex through propensity score matching with a matching tolerance of 0.5.

d *p-values were calculated using the rank–sum test or chi-square tests.*

### 3.2 Lymphocyte exhaustion and high CRP levels in adult COVID-19 patients, but not in children

**Figure 2:**
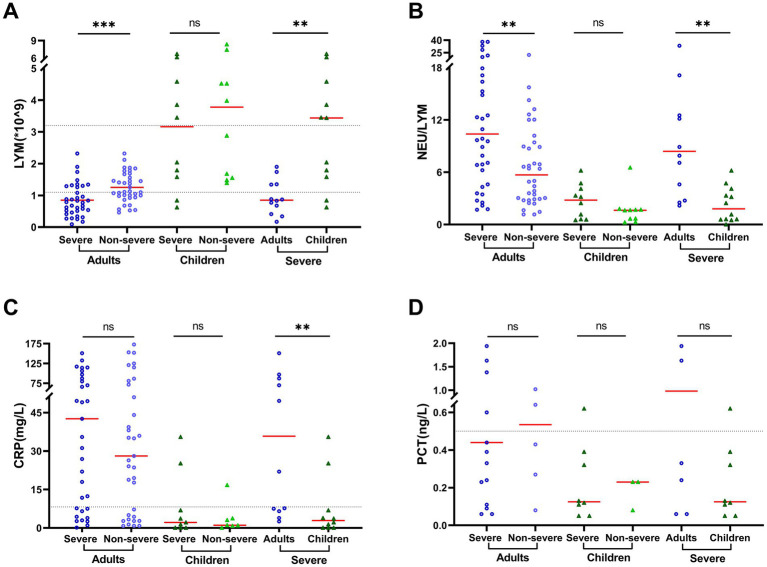
Clinical characteristics vary by age and patient outcome. Clinical measurements included **(A)** lymphocyte counts (LYM), **(B)** ratio of neutrophil vs. lymphocyte counts (NEU/LYM), **(C)** serum concentrations of C-reactive protein (CRP), and **(D)** serum concentrations of procalcitonin (PCT). The black dotted lines represent the reference range of 1.1–3.2 × 10^9^/L for LYM, 0.068–8.2 mg/L for CRP, and 0–0.5 ng/L for PCT. Data are presented as median and interquartile range (IQR) when they are not normally distributed or mean ± SD when normally distributed. The comparison was conducted by rank–sum test or *t*-test. *p < 0.05; **p < 0.01; ***p < 0.001; and ****p < 0.0001.

Dramatic decrease of lymphocyte accounts and increased ratio of neutrophil/lymphocytes (NEU/LYM) were observed in adult patients. A significant difference was found between severe and non-severe adult patients in the lymphocyte accounts (0.85 × 10^9^/L vs. 1.25 × 10^9^/L, *p* = 0.001) and NEU/LYM ratio (10.40 vs. 5.70, *p* = 0.003, Supplementary Table S2; Figure 2). Furthermore, the age-related difference was also obtained between adults and children with severe COVID-19 in the lymphocyte accounts (0.85 × 10^9^/L vs. 3.44 × 10^9^/L, *p* = 0.003) and NEU/LYM ratio (8.40 vs. 1.80, *p* = 0.002, Supplementary Table S2; Figure 2). In addition, a higher level of C-reactive protein (CRP), an inflammation biomarker, was observed in adult severe patients than in child severe patients (35.80 mg/L vs. 2.89 mg/L, *p* = 0.005), and in the adult severe patients than in non-severe adult patients (42.60 vs. 28.10, *p* = 0.456). However, the difference was not statistically significant (Supplementary Table S2; Figure 2). Other blood biochemical results showed substantial differences between severe adult patients and pediatric patients, but the levels of these biomarkers were still within normal ranges (Supplementary Table S2).

### 3.3 Higher levels of proinflammatory cytokines in BALF in adult COVID-19 patients than in children

**Figure 3:**
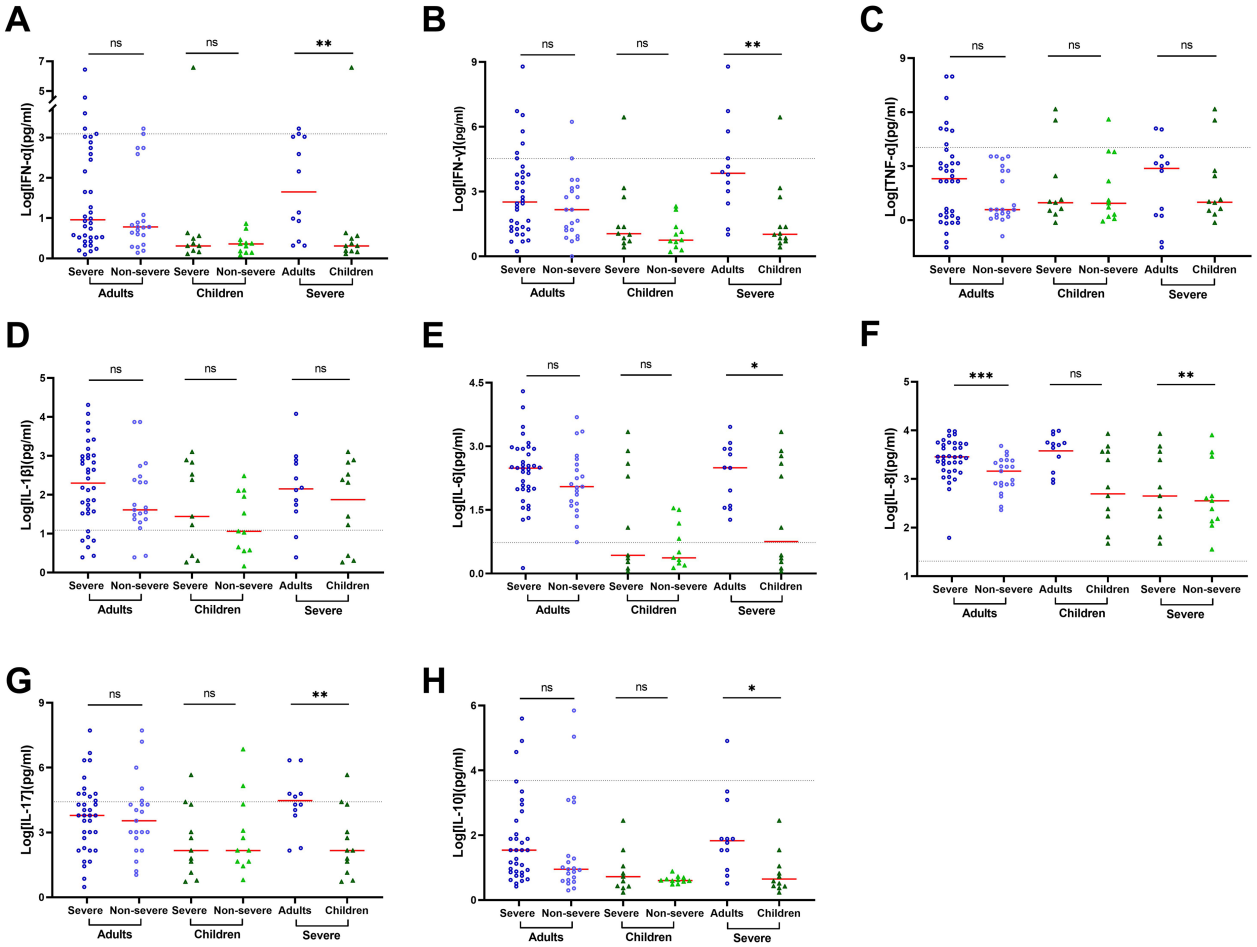
Cytokine concentrations vary by age and patient outcome in BALF of COVID-19 patients. Cytokine concentrations in BALF samples obtained during disease progression were determined using a 12-item cytokine detection kit for IFN-*α* **(A)**, IFN-*γ* **(B)**, TNF-α **(C)**, IL-1β **(D)**, IL-6 **(E)**, IL-8 **(F)**, IL-17 **(G)**, and IL-10 **(H)**. The black dotted lines represent the reference range, which is IFN-α ≤ 8.5 pg./mL, IFN-γ ≤ 23.1 pg./mL, TNF-α ≤ 16.5 pg./mL, IL-1β ≤ 12.4 pg./mL, IL-6 ≤ 5.4 pg./mL, IL-8 ≤ 20.6 pg./mL, IL-17 ≤ 21.4 pg./mL, and IL-10 ≤ 12.9 pg./mL, respectively. The concentrations in each group were compared by rank–sum test or *t*-test. Data are presented as expressed as median and interquartile range (IQR) when they are not normally distributed or mean ± SD when they were normally distributed. *p < 0.05, **p < 0.01, ***p < 0.001, and ****p < 0.0001.

Compared to non-severe adult COVID-19 patients, severe adult patients exhibited dramatically increased secretion of Th1 proinflammatory cytokines, in particular, IL-1β (40.79 vs. 207.84), IL-6 (112.90 vs. 304.15), and IL-8 (1454.66 vs. 2829.79, *p* = 0.001) in BALF (Supplementary Table S3). However, only IL-8 moderately increased in severe pediatric patients (451.62) and non-severe pediatric patients (355.87, Supplementary Table S3, Figure 3). Furthermore, higher levels of cytokines were observed in BALF in adults compared to children with severe COVID-19 (Supplementary Table S3; Figure 3). Specifically, a significant difference was observed for IL-6 (312.57 vs. 7.43, *p* = 0.05), IL-8 (4949.34 vs. 2054.95, *p* = 0.008), IL-5 (15.12 vs. 1.84, *p* = 0.013), IL-10 (3.56 vs. 1.57, *p* = 0.022), IL-17 (22.43 vs. 4.48, *p* = 0.007), IFN-*γ* (14.44 vs. 2.04, *p* = 0.004), and IFN-*α* (3.34 vs. 1.24, *p* = 0.005). However, no significant difference was observed for IL-1β (142.14 vs. 116.23, *p* = 0.371) between adults and children with severe COVID-19. In contrast, the level of TNF-α was higher in adults than in children (7.41 vs. 2.00) but did not reach statistical significance (*p* = 0.644).

### 3.4 Higher levels of cytokines in serum in children than in adults

**Figure 4:**
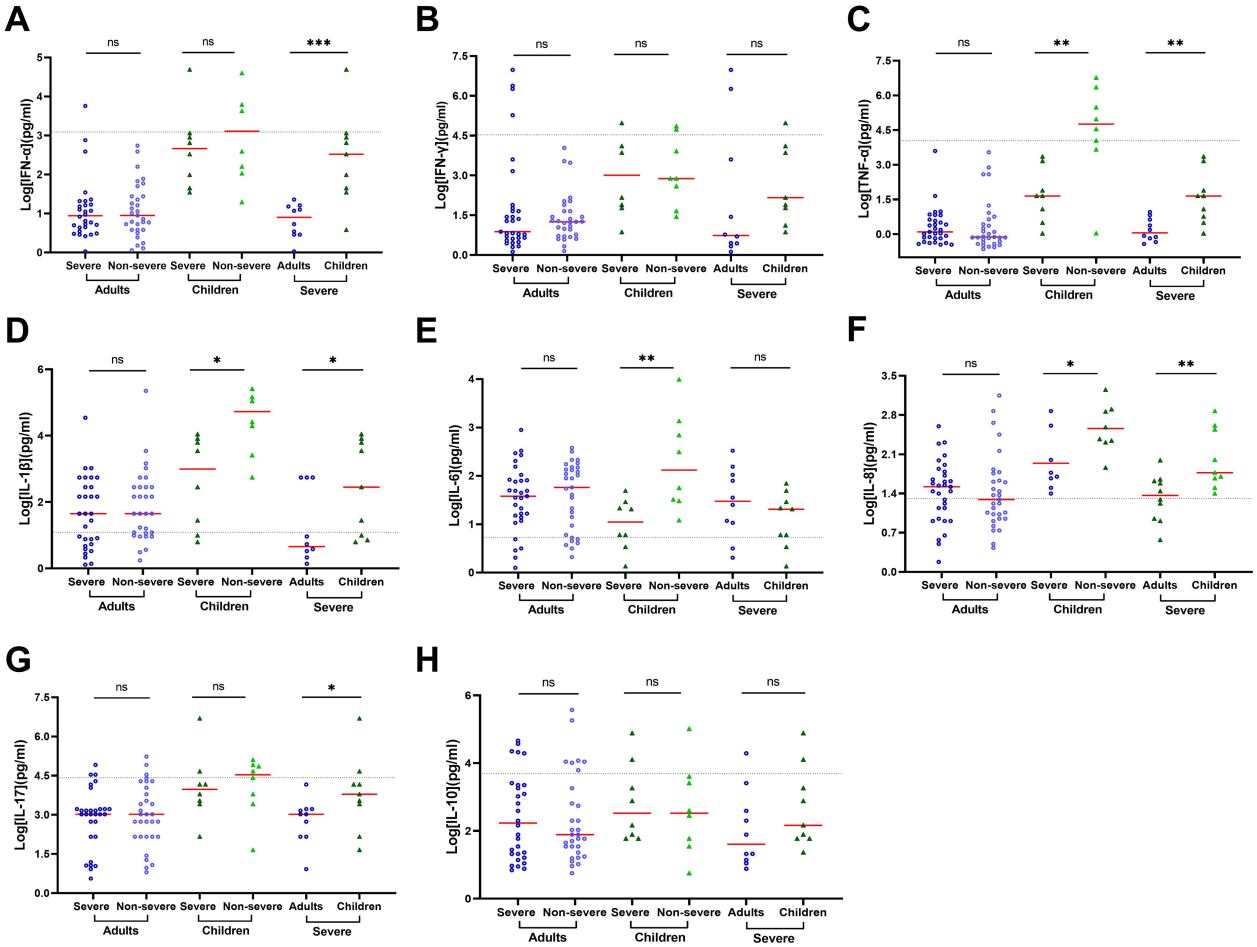
Cytokine concentrations vary by age and patient outcome in serum of progressive COVID-19 patients. Cytokine concentrations in serum samples obtained during disease progression were determined using a 12-item cytokine detection kit for IFN-α **(A)**, IFN-γ **(B)**, TNF-α **(C)**, IL-1β **(D)**, IL-6 **(E)**, IL-8 **(F)**, IL-17 **(G)** and IL-10 **(H)**. The black dotted lines represent the reference range, which is IFN-α ≤ 8.5 pg./mL, IFN-γ ≤ 23.1 pg./mL, TNF-α ≤ 16.5 pg./mL, IL-1β ≤ 12.4 pg./mL, IL-6 ≤ 5.4 pg./mL, IL-8 ≤ 20.6 pg./mL, IL-17 ≤ 21.4 pg./mL, and IL-10 ≤ 12.9 pg./mL, respectively. The concentrations in each group were compared by rank–sum test or *t*-test. Data are presented as expressed as median and interquartile range (IQR) when they are not normally distributed or mean ± SD when they were normally distributed. *p < 0.05; **p < 0.01; ***p < 0.001; and ****p < 0.0001.

SARS-CoV-2 did not induce significant IFN-α response or secretion of proinflammatory cytokines in the serum in the adult COVID-19 patients though the levels of IL-6, IL-8, IL-17, and IL-10 moderately increased (Supplementary Table S4). In addition, the ratio of cytokine concentrations between adult patients with or without severe COVID-19 ranged from 0.66 to 1.68 with an average of 1.05 ± 0.26 (Supplementary Table S4), indicating no significant association of blood cytokines with the severity of COVID-19 in adult patients. In contrast, a relatively higher level of blood cytokines was observed in pediatric patients than in the adults, especially for IFN-*α*, IFN-*γ*, TNF- α, IL-1β, IL-6, IL-8, and IL-17 (Supplementary Table S4). Of note, a relatively lower level of cytokines was observed in severe pediatric patients than in non-severe pediatric patients. The corresponding ratios were 0.69 for IFN-α, 0.11 for TNF-α (*p* = 0.009), 0.32 for IL-1β (*p* = 0.012), 0.07 for IL-6 (*p* = 0.006), 0.18 for and IL-8 (*p* = 0.036, Supplementary Table S4; Figure 4). These results indicated stronger cytokine expression in serum in pediatric patients (especially non-severe pediatric patients) than in adults.

### 3.5 Higher levels of cytokines in BALF than in serum

The levels of cytokines in BALF and serum were compared in COVID-19 patients whose BALF and serum samples were available. Significantly higher levels of IL-1β, IL-6, and IL-8 were observed in BALF than in serum in adult patients and pediatric severe patients, but not in non-severe pediatric patients (Table 2). For severe adult patients, the corresponding ratios of cytokines in BALF to serum were 70.94 for IL-1β (*p* < 0.001), 8.12 for IL-6 (*p* < 0.001), 93.58 for IL-8 (*p* < 0.001), 4.04 for TNF-*α* (*p* < 0.001), and 3.03 for IFN-*γ* (*p* = 0.010) while for non-severe adult patients, the corresponding ratios were 9.74 for IL-1β (*p* = 0.001), 1.47 for IL-6 (*p* = 0.010), and 130.19 for IL-8 (*p* = 0.001; Table 2). Interestingly, only a low level of IFN-α was detected, and there was no significant difference between BALF and serum in both severe patients (1.79 vs. 1.97, *p* = 0.421) and non-severe patients (1.75 vs. 1.78, *p* = 0.668). In contrast, a higher level of IL-10 was observed in serum than in BALF in both severe patients (4.10 vs. 2.91, *p* = 0.050) and non-severe patients (3.56 vs. 1.99, *p* = 0.006; Table 2). For severe pediatric patients, the corresponding ratios of cytokines in BALF to serum were 37.52 for IL-1β, 9.51 for IL-6, and 7.66 for IL-8 (*p* = 0.0015). In contrast, other cytokines, including IL-17 and IL-10, were relatively higher in serum than in BALF. Interestingly, except for IL-8, relatively higher levels of cytokines were observed in serum than in BALF in non-severe pediatric patients.

**Table 2:** Comparison of cytokine levels between the BALF and serum samples from COVID-19 patients.^a^

Cytokine^b^	Severe COVID-19 adults				Non-severe COVID-19 adults				Severe COVID-19 children				Non-severe COVID-19 children			
	BALF (*n* = 32)	Serum (*n* = 32)	Ratio^c^	*p*-value^d^	BALF (*n* = 20)	Serum (*n* = 20)	Ratio	*p*-value	BALF (*n* = 9)	Serum (*n* = 9)	Ratio	*p*-value	BALF (*n* = 22)	Serum (*n* = 22)	Ratio	*p*-value
IFN-α	1.79 (1.43, 4.14)	1.93 (1.49, 2.46)	0.93	0.421	1.75 (1.53, 5.09)	1.78 (1.49, 3.48)	0.98	0.668	1.24 (1.13, 1.51)	5.75 (3.02, 8.04)	0.22	0.110	1.26 (1.13, 1.35)	8.37 (3.71, 13.89)	0.15	**<0.001**
IFN-γ	5.46 (2.45, 13.02)	1.80 (1.48, 3.33)	3.03	**0.010**	4.48 (2.35, 10.33)	2.39 (2.00, 3.08)	1.87	**0.007**	1.79 (1.58, 2.56)	4.48 (2.78, 24.30)	0.40	0.086	1.72 (1.35, 2.05)	5.06 (2.47, 15.40)	0.34	**<0.001**
TNF-α	4.48 (1.14, 13.53)	1.11 (0.88, 1.54)	4.04	**<0.001**	1.58 (1.17, 6.68)	0.91 (0.82, 1.51)	1.74	**0.012**	2.03 (1.49, 6.07)	3.13 (1.55, 6.32)	0.65	0.953	1.30 (1.08, 2.10)	17.53 (1.49, 41.06)	0.07	**0.002**
IL-2	2.64 (1.92, 4.48)	2.27 (1.81, 3.08)	1.16	0.240	2.56 (2.04, 3.13)	2.15 (1.80, 2.67)	1.19	**0.038**	2.29 (1.93, 3.14)	3.40 (1.91, 3.80)	0.67	0.214	1.86 (1.67, 2.13)	2.93 (2.36, 5.04)	0.63	**<0.001**
IL-12	1.63 (1.42, 2.01)	1.57 (1.47, 1.76)	1.04	0.581	1.52 (1.43, 1.80)	1.66 (1.53, 1.77)	0.92	0.563	1.51 (1.46,1.58)	1.65 (1.47, 1.75)	0.92	0.214	1.52 (1.44, 1.60)	1.80 (1.61, 2.25)	0.84	**<0.001**
IL-4	1.56 (1.38, 1.87)	1.48 (1.24, 1.81)	1.05	0.166	1.60(1.46,1.90)	1.75 (1.37, 1.90)	0.91	0.563	1.44 (1.37, 4.00)	1.31 (1.23, 1.80)	1.10	0.594	1.47 (1.30, 1.80)	1.92 (1.66, 2.56)	0.77	**0.001**
IL-1β	207.84 (35.29, 1045.49)	2.93 (1.52, 5.46)	70.94	**<0.001**	43.62 (24.95, 207.07)	4.48 (1.99, 6.37)	9.74	**0.001**	204.84 (2.36, 728.87)	5.46 (1.90, 14.44)	37.52	0.086	10.65 (4.29, 43.26)	20.42 (5.94, 43.57)	0.52	0.592
IL-6	300.88 (97.02, 808.38)	37.03 (12.36, 82.92)	8.13	**<0.001**	110.51 (41.06, 254.32)	75.27 (18.76, 149.23)	1.47	**0.011**	194.04 (1.21, 680.92)	20.40 (4.72, 38.68)	9.51	0.139	2.07 (1.33, 3.37)	35.86 (16.47, 1393.98)	0.06	**<0.001**
IL-8	2829.79 (1482.64, 5387.29)	30.24 (8.33, 59.40)	93.58	**<0.001**	1592.28 (776.16, 2453.31)	12.23 (7.04, 41.93)	130.19	**0.001**	451.62 (116.65, 3652.29)	58.95 (38.95, 376.76)	7.66	**0.015**	209.46 (124.05, 505.46)	228.39 (118.61, 1043.29)	0.92	0.570
IL-17	13.81 (5.32, 27.05)	8.13 (4.48, 9.35)	1.70	**0.002**	13.60 (7.04, 30.17)	7.41 (4.48, 11.37)	1.84	0.084	4.48 (1.95,7.41)	13.81 (7.57, 21.62)	0.32	**0.028**	3.13 (1.74, 4.73)	20.42 (10.54, 26.21)	0.15	**<0.001**
IL-5	2.39 (1.76, 11.65)	2.26 (1.75, 3.98)	1.06	0.108	7.38 (1.82, 16.78)	3.27 (2.21, 6.28)	2.26	**0.007**	1.88 (1.50,7.81)	2.72 (2.02, 26.61)	0.69	0.214	1.52 (1.43, 1.64)	3.27 (1.99, 6.53)	0.46	**<0.001**
IL-10	2.91 (1.81, 6.38)	4.10 (2.36, 10.20)	0.71	**0.050**	1.99 (1.63, 2.68)	3.56 (2.38, 6.94)	0.56	**0.006**	1.65 (1.39,4.19)	4.48 (3.40, 13.35)	0.37	0.110	1.53 (1.43, 1.62)	4.59 (2.91, 8.20)	0.33	**<0.001**

a All the subjects were self-matched, and all the variables were expressed as the median and interquartile range (IQR) when they were not normally distributed data or mean ± SD when they were normally distributed data.

b The normal reference range is as follows: IL-1β ≤ 12.4 pg./mL, IL-2 ≤ 7.5 pg./mL, IL-4 ≤ 8.56 pg./mLL, IL-5 ≤ 3.1 pg./mL, IL-6 ≤ 5.4 pg./mL, IL-8 ≤ 20.6 pg./ml, IL-10 ≤ 12.9 pg./mL, IL-12 ≤ 3.4 pg./mL, IL-17 ≤ 21.4 pg./mL, TNF-α ≤ 16.5 pg./mL, IFN-γ ≤ 23.1 pg./mL, IFN-α ≤ 8.5 pg./mL.

c Ratio = median of BALF/median of serum.

d *p-Values were calculated using the rank–sum test or t-test. Bold p-values indicate statistically significant, that is, p < 0.05.*

## 4 Discussion

In this study, we aimed to simultaneously compare the inflammatory response profile in lung and blood among adults and children with severe and non-severe COVID-19 to identify the biomarkers that relate to the severity of COVID-19 and uncover the distinct mechanisms of pathogenesis caused by SARS-CoV-2 infection among adults and children. In our study, well-documented hallmarks of severe COVID-19, such as lymphopenia, a higher neutrophil-to-lymphocyte ratio (NEU/LYM), and the proinflammatory cytokine storm including IL-6, IL-1β, IL-8, and TNF-*α* were observed in adult COVID-19 patients rather than pediatric patients. These findings emphasize age-related distinct immune responses as major players in the pathogenesis of COVID-19 and key factors affecting the clinical outcome of SARS-CoV-2 infection.

Lymphocyte depletion, especially low CD3^+^ and CD3^+^CD8^+^ T cell counts is a common feature of adult COVID-19 patients, which may be the result of a large number of T cells migrating to the lungs and other inflammatory sites (Nasrollahi et al., 2023). Although the exact mechanism of lymphatic depletion in COVID-19 is not fully understood, it may involve the overexpression of the NKG2A receptor on CD8^+^ T cells since the NKG2A receptor is upregulated in COVID-19 patients, and decreases during recovery of COVID-19 (Sun et al., 2023). Furthermore, in COVID-19 patients, there is an observed upregulation in the expression of immune checkpoint molecules such as PD-1, CTLA-4, TIM-3, LAG-3, TIGIT, and VISTA. Interference with immune checkpoints may contribute to exacerbating lymphocyte depletion (Gurshaney et al., 2023). In addition, SARS-CoV-2 is known to impede antigen presentation by downregulating the expression of major histocompatibility complex (MHC) class I and II molecules, which hampers the activation of T cell-mediated immune responses, further complicating the immune system’s ability to combat the infection (Mohammed et al., 2022) effectively. Our results support the vital role and potential therapeutic targets of immune checkpoints in COVID-19. In contrast, pediatric patients displayed only moderate systemic inflammation without lymphopenia, which is consistent with the lack of proinflammatory cytokine storm in the lung. This indicates less susceptibility to hyperinflammation during SARS-CoV-2 infection in children, possibly due to differences in innate immune activation and cytokine regulation (Van De Garde et al., 2024).

Another significant difference between adults and pediatric COVID-19 patients is the level of CRP. During the course of COVID-19, cytokine storms lead to enhanced CRP production in adult patients. CRP is primarily regulated by IL-6 and IL-1β and can activate the complement system and potentially exacerbate inflammatory injury. Moreover, CRP-activated NLRP3 inflammasomes may precipitate complications, and lead to cell death (Maity et al., 2023). Although CRP is a non-specific biomarker of acute infections and elevates across diverse conditions, especially in bacterial infections (Giamarellos-Bourboulis et al., 2020), our results are consistent with previous studies to support it as a reliable biomarker to distinguish adults and children with COVID-19 (Rotulo and Palma, 2023). Augmented CRP is not only an early biomarker of SARS-CoV-2 infection but is also associated with the severity and prognosis of COVID-19.

Anti-viral infections are generally initiated by activating innate immune responses to produce interferons and proinflammatory cytokines to block infections (Mathew et al., 2020). IFN-*α* triggers the activation of interferon-stimulated genes (ISGs) in the epithelial cells, which is crucial for both innate and adaptive immune responses. Our results and previous studies indicated that during the early stage of SARS-CoV-2 infection, especially in severe adult COVID-19 patients, type I interferon response including IFN-*α* production and activity is markedly suppressed, which in turn facilitates persistent viral replication, excessive proinflammatory cytokine production, and systemic inflammation (Hadjadj et al., 2020), although excessive type I IFN response is a well-documented factor that can worsen COVID-19 by promoting hyperinflammation (Lee et al., 2020). Previous studies have shown that a delayed but enhanced IFN-I response during pathogenic coronavirus infection is critical for the development of ARDS and increases mortality (Kindler and Thiel, 2016; Channappanavar and Perlman, 2017). The timing and magnitude of IFN-I responses also play a crucial role in disease progression and outcome. However, pediatric COVID-19 patients usually exhibit higher IFN-*α* levels due to enhanced expression of RIG-I, MDA5, and toll-like receptors (TLRs 2/3/4/7/8) in the respiratory tract to trigger a robust and early antiviral response (Thorne et al., 2021). Furthermore, low levels of toll-like receptors (TLRs) and interferon regulatory factors (IRF) in children’s monocytes and dendritic cells result in relatively restrained activation of the IFN-I signaling pathway, which may reduce inflammatory responses and lower the severity of COVID-19. This restricted but effective IFN response in children may mitigate the cytokine storm and tissue damage, and decrease the likelihood of severe outcomes (Jia et al., 2024). Moreover, the combined elevation of IFN-*γ* and TNF-*α* can activate JAK/STAT1/IRF1 signaling pathway and promote nitric oxide production, which may lead to caspase-8/FADD-mediated inflammatory cell death (Dharra et al., 2023). Our results are consistent with previous studies and further confirm that in patients with severe COVID-19, decreased lung function is associated with elevated systemic levels of IFN-γ and TNF-α (Littlefield et al., 2022). Our results highlight the critical role of innate immune response during early SARS-CoV-2 infection and support the administration of antiviral therapy as soon as possible to eliminate or restrict viral replication.

Consistent with previous studies, we observed a dramatic increase of IL-6, IL-1β, IL-8, and TNF-*α* in BALF in adult COVID-19 patients, which is the main hyperinflammatory feature of severe COVID-19 patients while both IL-4 and IL-10 moderately increased to act as anti-inflammatory cytokines through a negative feedback mechanism (Xiong et al., 2020). Previous studies have found notable discrepancies in the intracellular gene expression between BALF and peripheral blood in COVID-19 patients (Manik and Singh, 2022). Our results indicated that SARS-CoV-2 infection results in enhanced secretion of Th1 proinflammatory cytokines, but a comparatively low secretion of Th2 anti-inflammatory cytokines in the BALF than in the serum of adult COVID-19 patients. The release of large amounts of cytokines and chemokines, particularly IL-6, IL-1β, IL-8, and TNF-α in the lung increases the expression of cellular adhesion molecules and VEGF, leading to an increase in pulmonary endothelial permeability, allowing viral dissemination and promoting the infiltration of neutrophils and inflammatory monocytes. Furthermore, the translocation of proinflammatory cytokines into the blood may lead to overactivation of Th1 cells and trigger systemic immune response (Manik and Singh, 2022). Conversely, children patients maintain cytokine levels within normal range and do not experience the cytokine storm typically seen in adult patients (Yuan et al., 2021). On the other hand, children are capable of sustaining appropriate but higher levels of inflammatory cytokines in the early stages of SARS-CoV-2 infection, which may aid in the more effective control of viral replication (Van De Garde et al., 2024). Of note, systemic immune responses are more often observed in children whose cytokine levels were observed to be higher in serum than in BALF. During acute SARS-CoV-2 infection, young children are relatively unaffected by the severe consequences reported in adults, but they are particularly vulnerable to multisystemic inflammatory syndrome in children (MIS-C). This may be due to immunodeficiency in children caused by Th2 polarization, which begins *in utero* and remains unchanged for the majority of the first decade of life (Hobbs et al., 2020). It should be noted that in children, early immune responses characterized by elevated levels of IL-17 can facilitate the quick clearance of viral infections (Rotulo and Palma, 2023), which is similar to our findings that serum IL-17 levels were higher in children than adults. The age-related immune polarization may explain why children are less susceptible to severe pulmonary complications but more prone to MIS-C (Wimmers et al., 2023). The significant difference in immune responses between adults and children could help to provide age-specific therapeutic strategies. For adults, interventions targeting cytokine storm are crucial to mitigate severe outcomes of COVID-19. Agents such as IL-6 inhibitors and anti-IL-1β antibodies could attenuate excessive inflammation while promoting viral clearance (Tasoudis et al., 2022).

Our study had several limitations. First, the participants were all hospitalized patients. We did not include people with mild COVID-19. Therefore, the results cannot be expanded directly to all SARS-CoV-2 infections. Second, we only analyzed cytokine profiles, but not lymphocyte subsets. Our results did not reflect the whole picture of abnormal immune responses in COVID-19 patients. Third, a single-center study with a small sample size of pediatric COVID-19 patients may limit the ability to identify the difference in cytokine levels between severe and non-severe pediatric patients. Additionally, our study did not include a control group of healthy subjects. A comparison with healthy controls could provide the baseline cytokine levels and strengthen the interpretation of the immune dysregulation in COVID-19 patients.

## 5 Conclusion

The severity of COVID-19 in adult patients is mainly caused by a deficiency in the innate immune response, which fails to control SARS-CoV-2 infection and replication, as well as overactivated immune response that results in cytokine storm in the lungs. In contrast, pediatric patients typically exhibit a moderate systemic immune response and a less cytokine storm in the lungs. The age-related differences uncover the different mechanisms related to the pathogenesis, prognosis, and treatment among adults and children with COVID-19. Cytokine profiles in COVID-19 patients may also offer new insights into the mechanism of long COVID-19 and facilitate the development of interventional measures to modulate immune responses and suppress inflammation in critically ill patients.

## Data Availability

The original data presented in the study are included in the article/supplementary material; further inquiries can be directed to the corresponding author.
