# Supplementary material for: Age-related immune response disparities between adults and children with severe COVID-19: a case–control study in China
Source: Front Microbiol. 2025 Feb 4;16:1525051. doi: 10.3389/fmicb.2025.1525051 (PMC11832681; doi:10.3389/fmicb.2025.1525051)
Supplement: Supplementary file 1 [file Table_1.docx]

**Supplementary materials**

**Table S1. Demographics and clinical features of adults and children with COVID-19.**

|  | **Adults(age≥18, n=86)** | **Children(age＜18, n=40)** | ***P* value^a^** |
| --- | --- | --- | --- |
| **Age (years, medium, IQR)** | 59.00(48.75,70.25) | 3.00(0.77,6.00) | **<0.001** |
| **Sex (n, %)** |  |  | 0.418 |
| Male | 60(69.8) | 25(62.5) |  |
| Female | 26(30.2) | 15(37.5) |  |
| **Antiviral treatment (n, %)** | 19(22.1) | 0(0) | **0.001** |
| **Mechanical ventilation (n, %)** | 7(8.1) | 0(0) | 0.063 |
| **Length of hospitalization (days, medium, IQR)** | 10.00(6.00,16.00) | 6.00(5.00,7.00) | **<0.001** |
| **Underlying chronic diseases (n, %)** |  |  | **<0.001** |
| Diabetes mellitus | 15(17.4) | 0(0) |  |
| Hypertension | 24(27.9) | 0(0) |  |
| Cerebral or cardiac vascular disease | 19(22.1) | 0(0) |  |
| Maiignant tumor | 16(18.6) | 0(0) |  |
| COPD | 2(2.3) | 0(0) |  |
| **Vaccination (n, %)**^b^ |  |  | **<0.001** |
| Unvaccinated | 19(30.2) | 25(67.6) |  |
| Incomplete vaccination | 0(0) | 0(0) |  |
| Full vaccination | 5(7.9) | 12(32.4) |  |
| Booster vaccination | 39(61.9) | 0(0) |  |
| **Outcome (n, %)** |  |  | **0.018** |
| Recovery | 75(87.2) | 40(100) |  |
| Death | 11(12.8) | 0(0) |  |

^a^P values were calculated using rank sum test or chi-square tests. Bold p value indicate statistically significant, i.e., p < 0.05.

^b^There were 3 samples with unknown vaccination status and 28 samples with unknown number of vaccinations. Partial vaccination: not fully vaccinated;full vaccination: full vaccination; booster vaccination: a booster dose was given on top of the full dose of vaccine.

**Table S2. Blood components and biochemical analysis results in the matched patients with COVID-19****^a^**

| **Biomarkers** | **Adults with COVID-19** | | | **Children with COVID-19** | | | **Severe COVID-19 patients** | | |
| --- | --- | --- | --- | --- | --- | --- | --- | --- | --- |
|  | **Severe (n=36)** | **Non-severe (n=36)** | **P value** | **Severe (n=11)** | **Non-severe (n=11)** | **P value** | **Adults(n=12)** | **Children(n=12)** | **P value^b^** |
| **D-dimer** | 0.54 (0.27, 0.86) | 0.40 (0.20, 3.53) | 0.971 | 0.25 (0.14, 0.47) | 0.79 (0.25, -) | 0.329 | 0.55 (0.22, 1.49) | 0.25 (0.14, 0.47) | 0.205 |
| **ALT** | 30.65 (15.23, 51.58) | 19.60(14.10, 31.75) | 0.100 | 22.90 (15.40, 35.88) | 17.60 (13.53, 19.92) | 0.082 | 42.15 (12.80, 80.68) | 20.80 (14.20, 34.90) | 0.255 |
| **AST** | 25.00 (19.60, 41.90) | 25.20 (18.33, 39.85) | 0.706 | 44.53 ± 22.21 | 29.99 ± 7.75 | 0.151 | 26.50 (17.38, 39.43) | 39.30 (24.60, 56.50) | 0.196 |
| **ALB** | 30.48 ± 5.37 | 34.05 ± 6.23 | **0.011** | 41.24 ± 3.62 | 43.45 ± 3.70 | 0.151 | 32.33 ± 5.41 | 41.46 ± 3.51 | **＜0.001** |
| **BUN** | 6.31 (4.64, 9.09) | 4.94 (3.68, 7.44) | **0.037** | 3.43 ± 0.80 | 3.87 ± 1.01 | 0.199 | 5.95 (3.97, 7.99) | 3.55 (2.57, 3.86) | **0.001** |
| **CR** | 69.60 (56.20, 88.58) | 68.65 (54.70, 85.5) | 0.783 | 29.44 ± 8.76 | 29.92 ± 10.42 | 0.880 | 63.40 (54.98, 79.05) | 29.50 (23.50, 36.70) | **＜0.001** |
| **UA** | 237.35 (183.58, 277.20) | 290.55 (242.95, 338.03) | **0.012** | 247.25 ± 85.20 | 254.48 ± 60.80 | 0.821 | 222.70 ± 72.92 | 241.56 ± 83.00 | 0.568 |
| **CRP** | 42.60(7.54, 106.00) | 28.10(4.40, 81.00) | 0.456 | 2.14 (0.09, 16.02) | 1.06 (0.81, 3.71) | 0.672 | 35.80 (6.81, 94.63) | 2.89 (0.09, 11.45) | **0.005** |
| **LDH** | 264.00 (199.05, 374.45) | 209.80 (170.13, 303.50) | 0.142 | 299.55 (245.30, 540.35) | 266.70 (228.50, 287.10) | 0.112 | 202.30 (159.45, 338.50) | 296.90 (233.90, 538.20) | **0.044** |
| **CK** | 57.10 (34.50, 96.55) | 45.40 (27.63, 98.65) | 0.363 | 99.00(40.50, 370.20) | 92.20(39.23, 125.15) | 0.406 | 39.50 (33.05, 104.40) | 96.30 (41.40, 291.50) | 0.087 |
| **CK-MB** | 11.00 (6.70, 14.95) | 9.60 (6.40, 12.00) | 0.210 | 23.29 ± 8.66 | 19.24 ± 5.32 | 0.545 | 7.78 ± 3.19 | 22.91 ± 8.31 | **＜0.001** |
| **WBC** | 8.23 (7.00, 12.30) | 9.07 (5.91, 11.75) | 0.822 | 10.74 (6.98, 12.99) | 10.63 (4.59, 13.44) | 0.821 | 7.82 (5.97, 11.37) | 10.45 (6.09, 12.39) | 0.424 |
| **LYM** | 0.85 ± 0.52 | 1.25 ± 0.47 | **0.001** | 3.16 ± 2.15 | 3.78 ± 2.51 | 0.705 | 0.85 (0.48, 1.35) | 3.44 (1.58, 4.58) | **0.003** |
| **NEU** | 6.66 (4.79, 11.43) | 7.07 (4.25, 9.57) | 0.408 | 2.67 ± 1.97 | 4.51 ± 2.95 | 0.450 | 6.16 (4.79, 9.74) | 3.84 (3.04, 5.66) | 0.124 |
| **NEU/LYM** | 10.40 (5.02, 19.34) | 5.70 (2.91, 9.31) | **0.003** | 2.81 (0.58, 4.25) | 1.63 (0.56, 1.72) | 0.290 | 8.40 (3.22, 15.97) | 1.80(0.57, 3.90) | **0.002** |
| **PCT** | 0.44 (0.17, 4.40) | 0.54(0.22, 1.27) | 0.889 | 0.13 (0.07, 0.37) | 0.23(0.08, -) | 1.000 | 0.98 (0.11,5.63) | 0.13 (0.07, 0.37) | 0.115 |
| **PLT** | 289.39 ± 144.02 | 290.94 ± 135.40 | 0.962 | 435.10 ± 217.12 | 391.60 ± 184.46 | 0.705 | 233.50 (177.25, 355.00) | 366.00 (273.00, 559.00) | 0.056 |

^a^All the variables were expressed as median and interquartile range (IQR) except for ALB and lymphocyte, which were expressed as mean ± SD since they were normally distributed data.

^b^P values were calculated using rank sum test or t test.

**Table S3. Cytokine levels in BALF of the matched patients with progressive COVID-19^a^**

| **Cytokines**^b^ | **Adults with COVID-19** | | | | **Children with COVID-19** | | | | **Severe COVID-19 patients** | | | |
| --- | --- | --- | --- | --- | --- | --- | --- | --- | --- | --- | --- | --- |
|  | **Severe (n=36)** | **Non-severe (n=36)** | **Ratio** | **P value** | **Severe (n=11)** | **Non-severe (n=11)** | **Ratio** | **P value** | **Adults(n=12)** | **Children(n=12)** | **Ratio** | **P value^c^** |
| **IFN-α** | 1.94(1.43,6.52) | 1.72(1.39,4.08) | 1.13 | 0.472 | 1.24(1.12,1.47) | 1.28(1.10,1.38) | 0.97 | 0.895 | 3.34 (1.48, 8.13) | 1.24 (1.12, 1.45) | 2.69 | **0.005** |
| **IFN-γ** | 5.75(2.45,14.75) | 4.48(2.12,9.14) | 1.28 | 0.233 | 2.07(1.63,6.68) | 1.68(1.35,2.56) | 1.23 | 0.224 | 14.44 (6.13, 47.24) | 2.04 (1.67, 5.65) | 7.08 | **0.004** |
| **TNF-α** | 4.97(1.14,14.20) | 1.50(1.19,7.41) | 3.31 | 0.309 | 1.96(1.25,5.46) | 1.92(1.14,13.81) | 1.02 | 0.974 | 7.41 (1.19, 10.94) | 2.00 (1.30, 6.38) | 3.71 | 0.644 |
| **IL-2** | 2.72(1.92,5.46) | 2.56(1.89,3.13) | 1.06 | 0.412 | 2.29(1.80,2.72) | 1.87(1.62,2.22) | 1.22 | 0.115 | 5.46 (2.17, 7.94) | 2.24 (1.87, 2.68) | 2.44 | 0.083 |
| **IL-12** | 1.67(1.43,2.06) | 1.51(1.40,1.74) | 1.11 | 0.148 | 1.53(1.47,1.88) | 1.45(1.42,1.73) | 1.06 | 0.622 | 1.87 (1.37, 2.32) | 1.52 (1.46, 1.81) | 1.23 | 0.236 |
| **IL-4** | 1.56(1.38,1.90) | 1.57(1.44,1.87) | 0.99 | 0.914 | 1.52(1.36,1.96) | 1.45(1.34,1.82) | 1.05 | 0.490 | 1.69 (1.41, 1.96) | 1.48 (1.36, 1.90) | 1.14 | 0.488 |
| **IL-1β** | 207.84(33.99,1017.70) | 40.79(21.38,266.90) | 5.10 | 0.081 | 27.62(1.99,681.57) | 11.61(3.71,125.82) | 2.38 | 0.577 | 142.14 (41.87, 740.07) | 116.23 (2.17, 594.55) | 1.22 | 0.371 |
| **IL-6** | 304.15(97.02,864.94) | 112.90(42.20,437.46) | 2.69 | 0.165 | 2.72(1.31,388.08) | 2.36(1.54,15.06) | 1.15 | 0.577 | 312.57 (36.74, 864.94) | 7.43 (1.45, 536.28) | 42.07 | **0.050** |
| **IL-8** | 2829.79(1482.64,5288.51) | 1454.66(751.75,2420.22) | 1.95 | **0.001** | 451.62(62.91,3652.29) | 355.87(135.80,2862.73) | 1.27 | 0.767 | 4949.34 ± 3120.69 | 2054.95 ± 2662.01 | 2.41 | **0.008** |
| **IL-17** | 13.81(5.32,27.62) | 11.61(5.58,20.83) | 1.19 | 0.648 | 4.48(2.20,19.53) | 4.48(3.13,19.68) | 1.00 | 0.645 | 22.43 (14.46, 67.63) | 4.48 (2.43, 16.68) | 5.01 | **0.007** |
| **IL-5** | 2.72(1.80,14.75) | 8.13(1.90,15.92) | 0.33 | 0.268 | 1.80(1.49,2.39) | 1.64(1.43,2.19) | 1.10 | 0.450 | 15.12 (2.39, 50.19) | 1.84 (1.50, 2.30) | 8.22 | **0.013** |
| **IL-10** | 2.91(1.83,7.48) | 1.93(1.53,5.35) | 1.51 | 0.091 | 1.65(1.34,2.91) | 1.52(1.48,1.61) | 1.09 | 0.694 | 3.56 (2.15, 7.32) | 1.57 (1.34, 2.70) | 2.27 | **0.022** |

^a^All the variables were expressed as median and interquartile range (IQR) when they were not normally distributed data or mean ± SD when they were normally distributed data.

^b^The normal reference range is as follows: IL-1β≤12.4pg/mL, IL-2≤7.5pg/mL, IL-4≤8.56pg/mL, IL-5≤3.1pg/mL, IL-6≤5.4pg/mL, IL-8≤20.6pg/mL, IL-10≤12.9pg/mL, IL-12≤3.4pg/mL, IL-17≤21.4pg/mL, TNF-α≤16.5pg/mL, IFN-γ≤ 23.1pg/mL, 1FN-α ≤ 8.5pg/mL.

^c^P values were calculated using rank sum test or t test.

**Table S4. Cytokine levels in sera of the matched patients with progressive COVID-19^a^**

| **Cytokines**^b^ | **Adults with COVID-19** | | | | **Children with COVID-19** | | | | **Severe COVID-19 patients** | | | |
| --- | --- | --- | --- | --- | --- | --- | --- | --- | --- | --- | --- | --- |
|  | **Severe (n=36)** | **Non-severe (n=36)** | **Ratio** | **P value** | **Severe (n=11)** | **Non-severe (n=11)** | **Ratio** | **P value** | **Adults(n=12)** | **Children(n=12)** | **Ratio** | **P value^c^** |
| **IFN-α** | 1.96 (1.56, 2.50) | 1.93 (1.49, 2.72) | 1.02 | 0.927 | 6.39 (3.34, 8.19) | 9.22 (4.20, 21.65) | 0.69 | 0.462 | 1.88 (1.38, 2.28) | 5.75 (3.02, 8.04) | 0.33 | **0.001** |
| **IFN-γ** | 1.84 (1.53, 3.40) | 2.38 (1.65, 3.13) | 0.77 | 0.678 | 9.45 (3.48, 27.88) | 7.38 (3.86, 23.60) | 1.28 | 0.916 | 1.67 (1.34, 28.21) | 4.48 (2.78, 24.30) | 0.37 | 0.072 |
| **TNF-α** | 1.07 (0.88, 1.55) | 0.92 (0.78, 1.56) | 1.16 | 0.379 | 3.13(1.58,7.63) | 27.34(13.6,72.31) | 0.11 | **0.009** | 1.05 (0.86, 1.60) | 3.13 (1.55, 6.32) | 0.34 | **0.004** |
| **IL-2** | 2.28 (1.82, 3.13) | 2.07 (1.77, 2.56) | 1.10 | 0.275 | 3.56(2.19,3.84) | 2.93(1.97,7.12) | 1.22 | 0.833 | 1.92 (1.64, 3.04) | 3.40 (1.91, 3.80) | 0.56 | 0.178 |
| **IL-12** | 1.57 (1.47, 1.76) | 1.58 (1.42, 1.74) | 0.99 | 0.490 | 1.65(1.47,1.77) | 1.78(1.51,2.16) | 0.93 | 0.401 | 1.57 ± 0.15 | 1.70 ± 0.35 | 0.92 | 0.297 |
| **IL-4** | 1.54 (1.25, 1.82) | 1.37 (1.26, 1.79) | 1.12 | 0.888 | 1.44 (1.22, 1.82) | 1.83 (1.53, 3.19) | 0.79 | 0.083 | 1.21(1.10, 1.61) | 1.31 (1.23, 1.80) | 0.92 | 0.121 |
| **IL-1β** | 3.13 (1.59, 5.46) | 3.13 (2.08, 5.46) | 1.00 | 0.206 | 8.54 (2.17, 14.75) | 27.08 (12.87, 40.91) | 0.32 | **0.012** | 1.58 (1.22, 6.68) | 5.46 (1.90, 14.44) | 0.29 | **0.022** |
| **IL-6** | 38.23 (13.81, 83.37) | 57.69 (6.04, 148.01) | 0.66 | 0.531 | 13.22 (4.06, 26.96) | 181.13 (30.63, 1196.47) | 0.07 | **0.006** | 30.58 (8.77, 116.29) | 20.40 (4.72, 38.67) | 1.50 | 0.253 |
| **IL-8** | 32.85 (8.93, 61.56) | 19.53 (8.93, 57.69) | 1.68 | 0.588 | 54.81 (35.20, 328.73) | 307.78 (207.22, 783.18) | 0.18 | **0.036** | 23.58 (8.73, 42.39) | 58.95 (38.95, 376.76) | 0.40 | **0.007** |
| **IL-17** | 8.13 (4.48, 9.35) | 8.13 (4.48, 16.42) | 1.00 | 1.000 | 15.86 (10.89, 23.48) | 23.32 (11.44, 29.80) | 0.68 | 0.494 | 8.13 (4.48, 9.35) | 13.81 (7.57, 21.62) | 0.59 | **0.049** |
| **IL-5** | 2.27 (1.74 ,4.07) | 2.72 (1.93, 4.94) | 0.83 | 0.176 | 2.93 (2.38, 37.04) | 3.50 (2.28, 6.36) | 0.84 | 0.958 | 2.98 (2.07,5.21) | 2.72(2.02,26.61) | 1.10 | 0.806 |
| **IL-10** | 4.48 (2.50, 10.20) | 3.71 (2.56, 9.55) | 1.21 | 0.855 | 5.93 (3.48, 15.25) | 5.75 (3.03, 11.76) | 1.03 | 0.752 | 3.11 (2.17, 7.19) | 4.48 (3.40, 13.35) | 0.69 | 0.177 |

^a^All the variables were expressed as median and interquartile range (IQR) when they were not normally distributed data or mean ± SD when they were normally distributed data.^b^The normal reference range is as follows: IL-1β≤12.4pg/mL, IL-2≤7.5pg/mL, IL-4≤8.56pg/mL, IL-5≤3.1pg/mL, IL-6≤5.4pg/mL, IL-8≤20.6pg/mL, IL-10≤12.9pg/mL, IL-12≤3.4pg/mL, IL-17≤21.4pg/mL, TNF-α≤16.5pg/mL, IFN-γ≤ 23.1pg/mL, 1FN-α ≤ 8.5pg/mL.

^c^P values were calculated using rank sum test or t test.
